# W + Cu and W + Ni Composites and FGMs Prepared by Plasma Transferred Arc Cladding

**DOI:** 10.3390/ma14040789

**Published:** 2021-02-07

**Authors:** Jiří Matějíček, Jakub Antoš, Pavel Rohan

**Affiliations:** 1 Department of Materials Engineering, Institute of Plasma Physics, Czech Academy of Sciences, 18200 Prague, Czech Republic; 2 Faculty of Mechanical Engineering, Czech Technical University in Prague, 16607 Prague, Czech Republic; antos@vzuplzen.cz (J.A.); pavel.rohan@fs.cvut.cz (P.R.); 3 Research and Testing Institute, 30100 Pilsen, Czech Republic

**Keywords:** plasma-facing materials, W-based composites, PTA cladding

## Abstract

Tungsten-based materials are the most prospective candidates for plasma-facing components of future fusion devices, such as DEMO. W-based composites and graded layers can serve as stress-relieving interlayers for the joints between plasma-facing armor and the cooling or structural parts. Coating/cladding techniques offer the advantages of eliminating the joining step and the ability to coat large areas, even on nonplanar shapes. In this work, W + Cu and W + Ni composites were prepared by pulsed plasma transferred arc (PTA) cladding on several different substrates. Optimization of the process was carried out with respect to powder mixture composition and process parameters like arc current, plasma gas composition, and traverse velocity. Dense claddings of several millimeters thickness and various W content were achieved. Moreover, multilayers with W content gradually varying from 47 to 92% were formed. The structure, compositional profiles, and thermal properties of the claddings were characterized.

## 1. Introduction

Plasma-facing components (PFCs) for future fusion reactors will have to withstand extremely harsh conditions involving high heat fluxes (both steady-state and thermal shocks) and bombardment of plasma species (ions, electrons, neutral atoms, and high-energy neutrons) [1]. Tungsten is considered as the prime candidate material for these components, particularly for its refractory nature (high melting point and high strength at elevated temperatures), high resistance to sputtering, good thermal conductivity, etc., [2,3,4]. However, joining of tungsten to steel- or copper-based structural or cooling system presents a significant challenge. In particular, the large difference in thermal expansion coefficients and moduli of elasticity leads to high stresses at the interface upon thermal loading [5]. Possible joining technologies include direct bonding, solid-state bonding or brazing with discrete interlayers, and the use of graded interlayers consisting of only tungsten and steel or copper [5]. The use of additional materials is constrained by the limited choice of elements due to temperature limitations and the requirement for low activation, thermodynamic stability, high yield strength, etc., [5]. On the other hand, graded interlayers provide a smooth transition, thereby reducing the stress concentration compared to a sharp interface. For appropriate stress redistribution, a thickness of the order of millimeters is needed [6]. An overview of several prospective fabrication technologies, such as plasma spraying, laser cladding, hot pressing, and spark plasma sintering, with their characteristics, advantages, and limitations was provided in [7].

W-rich composites with small percentage of Ni + Fe or Ni + Cu, commonly called “tungsten heavy alloys”, are considered as possible plasma-facing materials as an alternative to tungsten. Their main advantage lies in higher ductility and machinability and lower cost (due to easier sintering) [8]. Potential drawbacks include significantly lower melting point and higher vapor pressure of the binder phase and lower thermal conductivity. When W + Ni + Fe samples were irradiated by deuterium plasma pulses, simulating off-normal events in ITER, they showed comparable cracking pattern to double forged tungsten but with a shallower damaged zone [9]. W + Ni + Fe tiles were tested in the divertor of ASDEX-U tokamak. Despite reaching the melting point of the binder phase in overloaded regions, the W skeleton retained its integrity, no increased influx of Ni or Fe into the plasma was observed, and overall these tiles behaved similarly to those of pure W [10].

Plasma transferred arc (PTA) cladding (sometimes also called PTA surfacing, hardfacing, or overlay welding) is a deposition technique that uses plasma to melt the filler material and substrate. This technology can be considered as a development of gas tungsten arc welding technique, while the plasma plume is constricted by water-cooled copper orifice and the tungsten rod is placed inside the torch. Filler material is fed into the plasma jet coaxially in the torch. After solidification, a new metallurgical bond is established between the deposit and the substrate. The thickness of the deposited layer varies between 0.5 and 10.0 mm [11,12], and the dilution (fraction of the substrate material in the weld) ranges from 3 to 10% for the majority of PTA applications [13,14]. PTA hardfacing is used for both new production and reparation of parts in valve, glass, oil, and other industrial fields [15,16,17]. Similar to plasma spraying [18,19], it is a direct deposition technique that eliminates the need for a further bonding step, offers the ability to coat large areas and the possibility of compositional gradation, but offers the additional advantage of depositing dense layers without pores and weakly bonded interfaces. The general benefit of gradually changing composition (so-called functionally graded material (FGM)) lies in the reduction of stress concentration that would otherwise occur at a sharp interface of two dissimilar materials upon thermal loading [6,20]. FGM formation by PTA and similar techniques was described in [21,22].

The present work explores the possibility of preparing W + Cu and W + Ni composites by PTA cladding. To our knowledge, this is the first application of the technique on materials with potential application in plasma-facing components of fusion devices. The objectives were to test the prospective advantages outlined above, i.e., formation of dense layers with significant material throughput without the need for additional bonding, and the compositional control. The latter included the demonstration of FGM formation and exploration of the limits in tungsten content. Basic optimization was carried out with respect to powder mixture composition and process parameters, and the claddings were characterized for their structure, compositional profile, and thermal properties.

## 2. Experimental

The cladding experiments were carried out on a PPC 250 R6 (KSK Česká Třebová, Czech Republic) weld surfacing equipment [23]. It is equipped with a water-cooled plasma torch with 4 mm W-rod, coaxial powder feeding, 4-axis torch positioning, and 2-axis workpiece positioning system and enables the feeding of two different powders at a preselected volumetric ratio, which are mixed in a mixing chamber before entering the plasma jet. As the plasma-forming gas, shrouding gas and carrier gas, various combinations of Ar and Ar with 2%, 6%, or 10% H_2_ were used. Besides other parameters, the composition of the gases also affected the heat input to the workpiece. Moreover, the reducing atmosphere helped to protect the clad area from contact with surrounding air to prevent oxidation. The parameters of the cladding process were varied within the following ranges: upper torch current 110–190 A, lower torch current 60–90 A, pulsation frequency 10.8–12.1 Hz, linear traverse velocity 0.4–0.8 mm/s, and swing 16 mm [24].

**Figure 1:**
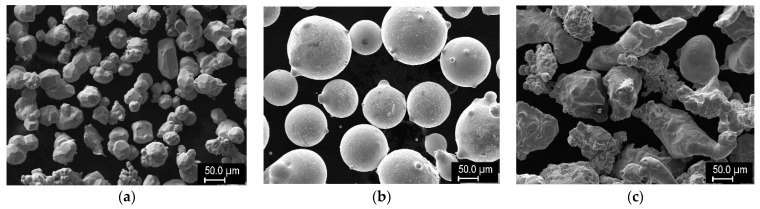
Morphologies of the feedstock powders: (**a**) W, (**b**) Ni alloy, (**c**) Cu.

Mixtures of the following powders were used as the feedstock: W (GTP, Towanda, PA, USA), Ni-340 PLK (LSN Diffusion, Llandybie, UK), and Cu (Stamont, Žilina, Slovakia). Their sizes and composition are presented in Table 1, and the representative morphologies are shown in Figure 1. These were mixed at selected ratios of powder volume. Stainless steel substrates (AISI 303) of 100 × 50 × 6 mm dimension were used for the experiments.

The following techniques were used for the characterization. The structure was observed on metallographically prepared cross sections by light microscopy (Neophot 32 and Zeiss Stemi DV4, Carl Zeiss, Göttingen, Germany) and by scanning electron microscopy (SEM; EVO MA 15, Carl Zeiss SMT, Oberkochen, Germany). On the SEM images, the percentage of W grains was determined by image analysis using ImageJ software (National Institute of Health, Bethesda, MD, USA). Local elemental composition was determined on the cross sections using energy-dispersive spectrometry (EDS; XFlash 5010, Bruker, Berlin, Germany) in the SEM. Surface composition of the claddings was determined by X-ray fluorescence spectrometry (XRF; DELTA, Olympus, Tokyo, Japan). Phase identification was performed with the help of X-ray diffraction (XRD) using a D8 Discover diffractometer (Bruker, Karlsruhe, Germany). Thermal conductivity was determined by laser flash technique at several temperatures between 20 and 400 °C using a LFA 1000 (Linseis, Selb, Germany) instrument. For this measurement, samples of ~5 × 10 × 10 mm dimension were used.

## 3. Results

### 3.1. W + Ni Cladding

In the first stage, several experiments were carried out focusing on the initial optimization with respect to the processing parameters (composition of the process gases, torch current and pulsation, powder feed rates, etc.). Only selected representative results are shown here, illustrating the salient characteristics of the claddings. Technological details are provided in [24].

**Figure 2:**
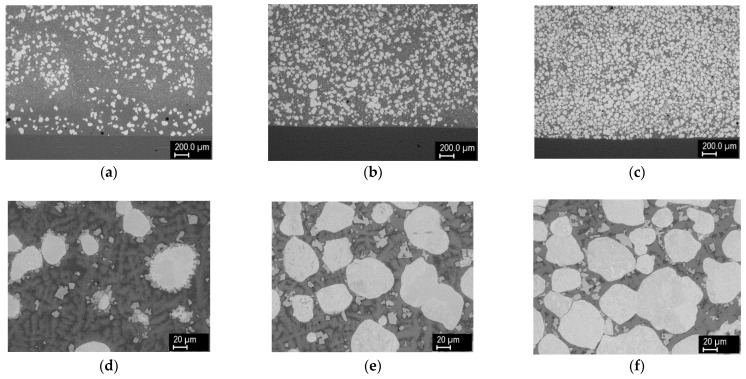

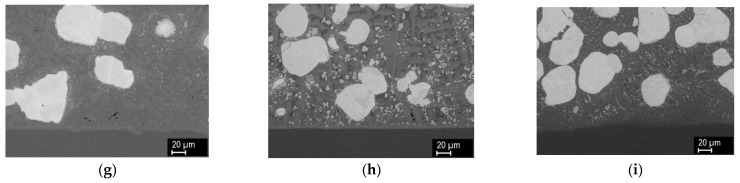
Cross sections of W–Ni claddings made from 25%, 50%, and 75% W mixtures (left, center, and right columns, respectively): (**a**–**c**) overview, (**d**–**f**) detail from the middle, (**g**–**i**) detail near the substrate. In this and the following figures, the substrate is the homogeneous region at the bottom of the respective images, free from tungsten grains (which is the lighter phase).

**Figure 3:**
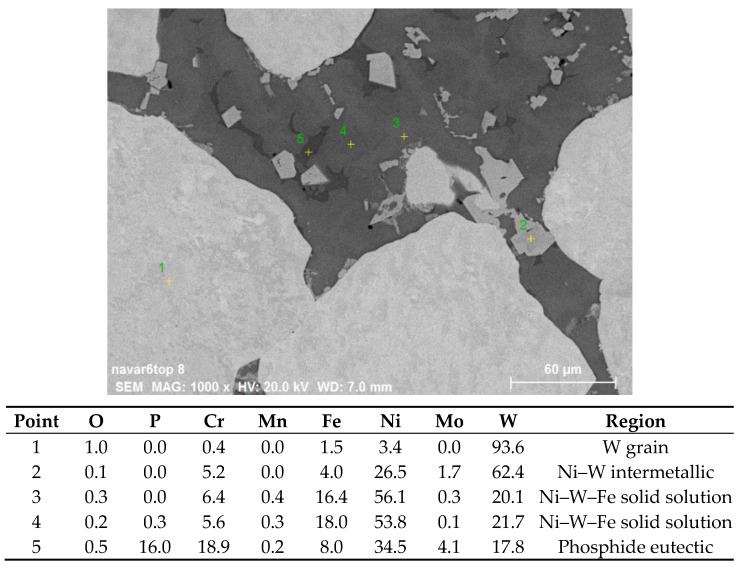
Results of local energy-dispersive spectrometry (EDS) analysis on the cross section of 75% W sample.

Figure 2 shows the cross sections of claddings formed from powder mixtures containing 25%, 50%, and 75% W (by volume) with Ar + 2% H_2_ carrier and shrouding gases. Very dense layers were observed in all cases. The smooth and straight interface with the substrate indicated rather low weld penetration, which was desired. With increasing W percentage in the powder mixture, the W content in the claddings also increased; however, the percentages were somewhat lower, with analysis of the SEM images indicating W content of about 18%, 35%, and 59%. It should be noted that the image analysis considered only the W grains and did not take into account W present in the other phases (see below); this may explain the lower numbers. With increasing W content, the homogeneity of spatial distribution of the W grains also increased. In contrast to the feedstock with roughly equiaxial but angular grains, the W grains in the claddings had a rounded shape, indicating that at least partial melting had occurred during the process. Different shades of gray in the backscattered electron images indicated strong intermixing of the elements and the formation of new phases. Thus, local elemental analysis was carried out, and the result is presented in Figure 3. In the W grain (point 1), traces of Fe, Cr, and Ni were found owing to the diffusion at elevated temperature. The light gray regions with sharp interfaces (point 2) apparently represent an intermetallic phase containing majority of W with significant amounts of Ni, Fe, and Cr. In the 25% W cladding, these were concentrated primarily at the surface of the W particles, while for the higher W content claddings, they appeared throughout the matrix without preferential locations. With higher concentration of W particles, the diffusion paths for W were apparently short enough, so the intermetallic particles could form more or less everywhere. Points 3 and 4 represent regions without sharp boundaries and were thus considered Ni–W–Fe solid solutions with varying composition. While Ni was the major element, significant amounts of Fe and Cr from the substrate were still present. As the analysis was done near the top of the cladding, this indicated that the substrate material was able to penetrate throughout its thickness due to convection in the melt pool. This was also confirmed by XRF analysis on the surface (Table 2), which showed the presence of Cr, Mo, and Fe elements from the substrate. Point 5 in Figure 3 indicates a phosphide eutectic formed due to low solubility of P in the present metals (P came from both the substrate and the Ni-based powder).

**Figure 4:**
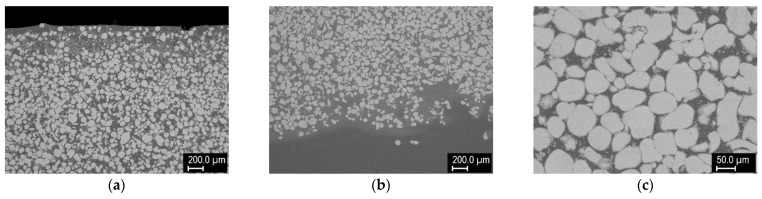
Cross sections of W–Ni cladding made from 95% W mixture: (**a**) overview near the top, (**b**) overview near the substrate, (**c**) detail from the middle of the cladding.

In the next stage, attempts were made to increase the W content in the claddings by increasing its percentage in the powder mixture. Merely increasing the W percentage in the feedstock led to porous claddings with insufficient melting, while increased heat input through increased torch current and the usage of Ar + 10% H_2_ as the carrier and shrouding gas led to excessive melting of the substrate. A number of parameter combinations were explored until a suitable combination of current pulsation, powder feed rate, and process gases was found, resulting in dense coatings with moderate interaction with the substrate. Despite increased W percentage (up to 95%), the content of the W grains in the claddings was still ~59%, i.e., the same as with the 75% W mixture (Figure 4).

**Figure 5:**
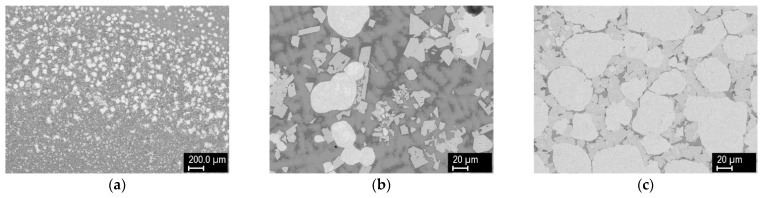
Cross sections of W–Ni cladding made from 50% W mixture on a graphite substrate: (**a**) overview, (**b**) detail from the middle of the cladding, (**c**) detail of a W-rich region.

**Figure 6:**
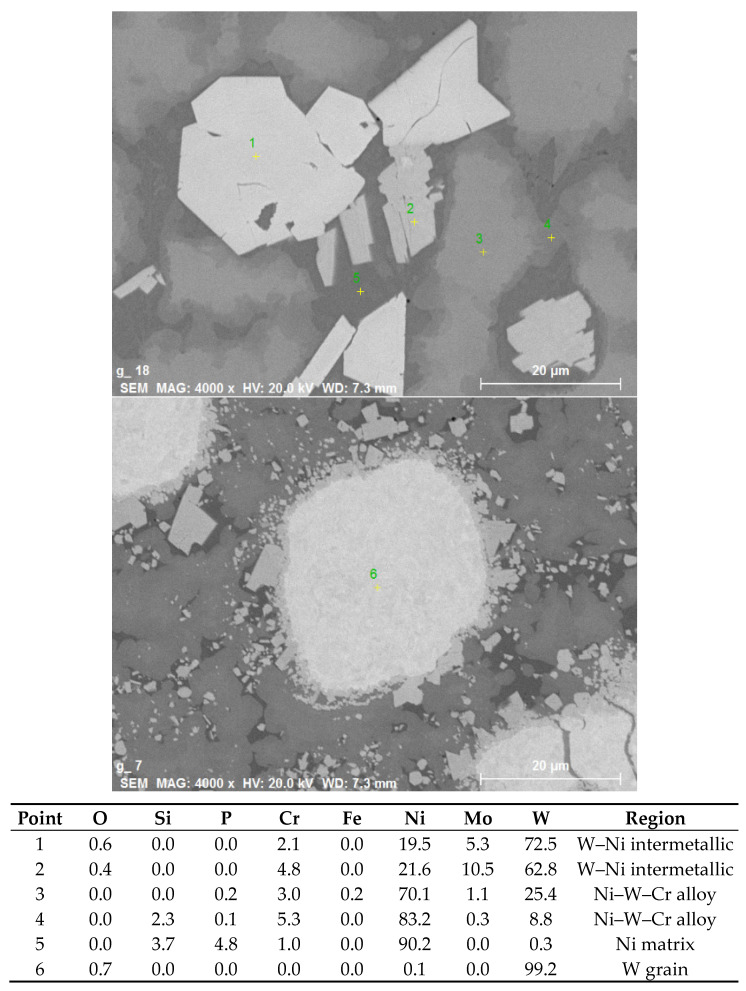
Results of local EDS analysis on the cross section of 50% W sample on a graphite substrate.

Figure 5 shows the cross section of claddings made on a non-melting substrate (graphite sheet). This combination was chosen to see whether the intermetallic formation could be avoided if steel is not present and whether dense layers can be formed without the aid of a melt pool from the substrate. Despite the absence of steel species, intermetallic formation was also observed. Representative compositions of the individual phases are shown in Figure 6. Compared to claddings on steel, more phases of varying composition were present, and there was negligible content of the matrix species in the W grains. The maximum content of W grains was about 39% in the layer from 50% W feedstock; higher W percentage layers were again porous.

### 3.2. W + Cu Cladding

**Figure 7:**
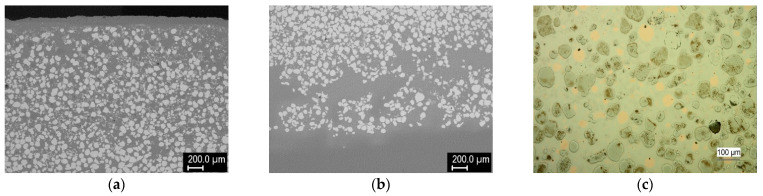
Cross sections of W–Cu cladding made from 75% W mixture on a steel substrate: (**a**) overview near the top, (**b**) overview near the substrate, (**c**) detail of a middle region (light microscopy for better distinction of the Cu grains in the steel matrix).

**Figure 8:**
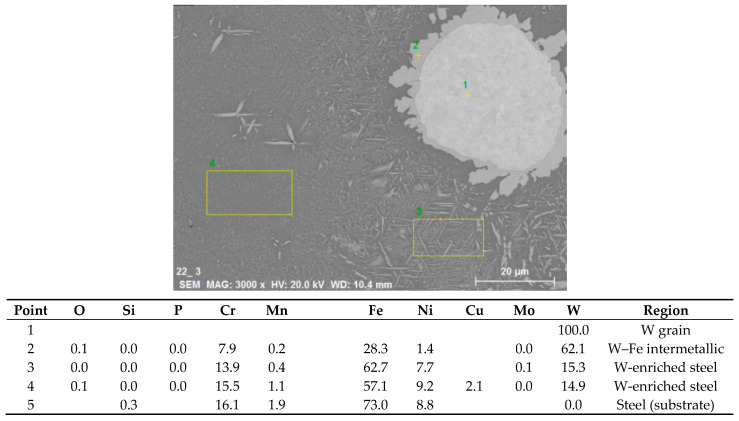
Results of local EDS analysis on the cross section of 75% W sample on a steel substrate.

Next, claddings from a mixture of W and Cu powders were produced. Representative structures of 75% W cladding on a steel substrate are shown in Figure 7. The structure looked similar to W + Ni claddings. However, contrary to the previous case where the matrix contained primarily Ni, for the W + Cu cladding, it consisted mostly of steel material with minority of copper grains dispersed throughout (Figure 7c). Additionally, about 100 μm layer of copper was observed at the top surface. Although steel has slightly lower density than copper, it might have increased due to tungsten dissolution in the steel matrix and helped the copper float to the surface (from the EDS data (Figure 8), the density of the W-enriched steel was estimated to be 10.2 g/cm^3^, while that of pure 303 steel and pure copper are 8 and 8.89 g/cm^3^, respectively [25]). Floating of a lighter phase to the surface was also observed in Gas Tungsten Arc Welding (GTAW) cladding [26]. Additionally, the copper influx from the cladding process, as well as its lower melting point, might have contributed to this phenomenon. Due to intense upward penetration of the substrate material and its interaction with the tungsten grains, Fe–W intermetallic was formed at the interfaces of tungsten grains with the steel-based matrix. XRD identified it as having the Fe_7_W_6_ structure, although the Fe/W ratio determined by EDS (Figure 8) was closer to the Fe_2_W type. The crystalline phase formation might have been affected by the alloying elements of the steel. In the steel matrix, quite a significant amount of dissolved W was observed, in addition to occasional intermetallic microlamellae (Figure 8). Few isolated pores were observed (Figure 7c), possibly as a result of copper overheating and boiling, although a grain pull-out during metallographic polishing cannot be ruled out. The high temperature in the melt pool (indicated by the rounded tungsten grains from the originally angular feedstock) was also likely responsible for copper loss through evaporation.

**Figure 9:**
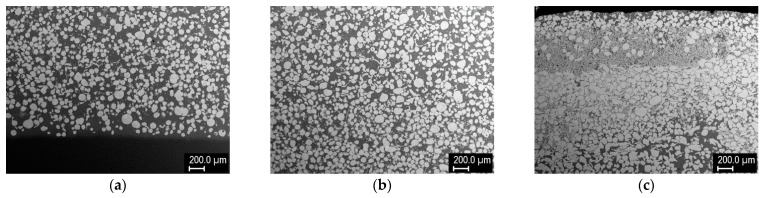
Cross sections of three-layer W–Cu cladding on a steel substrate: (**a**) one-layer (75% W), (**b**) two-layer (75% + 95% W), (**c**) three-layer (75% + 95% + 98% W).

**Figure 10:**
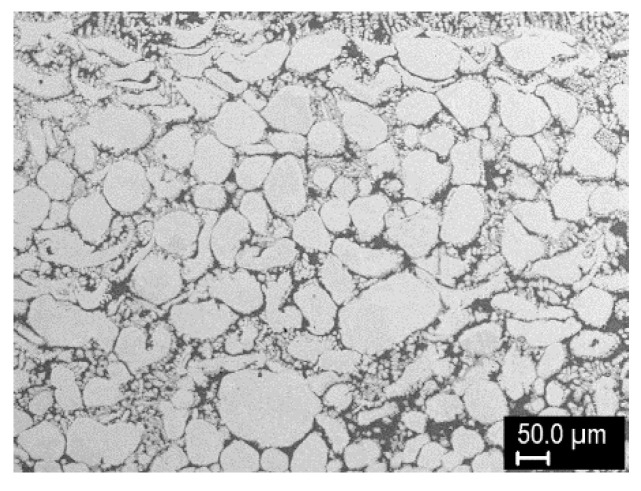
Detail of the W-rich region in the three-layer sample.

Finally, after another series of optimization experiments, a three-layer cladding was produced, with the aim of demonstrating FGM formation and maximization of the W content. The three layers were formed by successive deposition of 75% W, 95% W, and 98% W mixtures, with gradually shortening of the length of the clad. Then, single-, two-, and three-layer regions were analyzed, because the individual layers did not have distinct boundaries due to strong mutual interaction. These three regions were about 5.5, 7.4, and 8.9 mm thick. The cross sections are shown in Figure 9. The W content gradually increased toward the top surface, both from layer to layer and within the layers. According to image analysis, the W grain content was about 48% in layer 1, 65–75% in layer 2, and 72–92% in layer 3. The maximum content of W grains (92%) was reached just below the top surface, followed by a thin layer with dendritic structure and slightly decreased W content (~82%). The presence of this layer might be a result of the process dynamics (rise of the lighter species and descent of the heavier grains in the melt pool); it could be easily removed by grinding. Detail of the region richest in W is shown in Figure 10. Besides W grains, fine intermetallic dendrites growing into the matrix were observed. Thus, it can be concluded that tungsten constitutes the vast majority of this layer.

**Figure 11:**
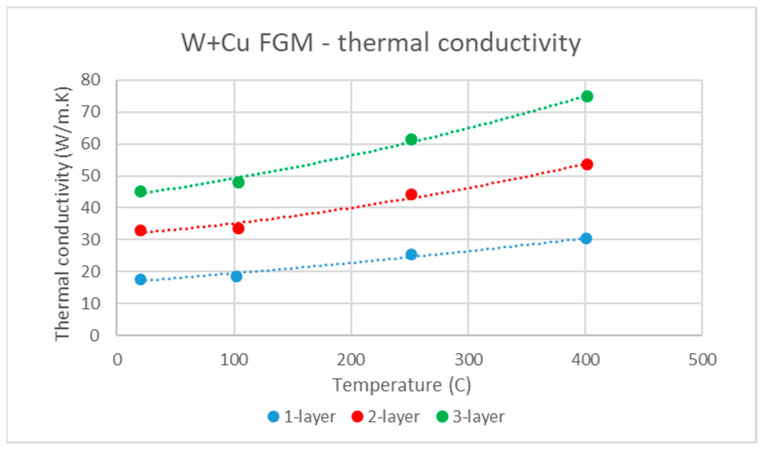
Temperature dependence of thermal conductivity of samples taken from the one-, two-, and three-layer regions of the functionally graded material (FGM).

The thermal conductivity of representative samples of these three layers is presented in Figure 11. It should be noted that 5 mm thick samples were cut out from the middle of each region and may therefore not correspond to a single composition. An increase of conductivity with overall W content was observed, as could be expected, as well as moderate increase with temperature. The values were markedly lower than those of both W and Cu due to significant presence of steel and Fe–W intermetallics in the claddings (typical thermal conductivities are 163 W/m·K for tungsten, 398 W/m·K for copper, 16 W/m·K for AISI 303 steel [25], and 25 W/m·K for Fe_7_W_6_ [27]). The thermal conductivity of the claddings was still comparable or higher than that of the main candidate structural material for PFCs, Eurofer97 steel, which is about 28 W/m·K [28].

## 4. Conclusions

In this paper, the results of pilot experiments with PTA deposition of W–Ni and W–Cu composites are presented. After a series of process optimization steps, several millimeters thick and fully dense layers with various W content were formed. The composition could be controlled by the feed rates of the individual powder ratio and other process parameters, such as torch current and composition of process gases. The W–Ni claddings consisted of W grains embedded in a matrix of Ni–Fe alloy(s) and W–Ni–Fe intermetallics, whose formation resulted from a strong interaction with the (molten) substrate material. The maximum achieved content of W grains was ~59 vol %. Deposition on a non-melting substrate—graphite—was proved feasible; the cladding still contained intermetallics (consisting of W, Ni, and Cr). In the claddings made from W–Cu powder mixture, the matrix contained more steel than Cu and also W–Fe intermetallics. As these are generally brittle and contribute to reduced thermal conductivity, they are considered undesirable. Their formation can be avoided by the use of feedstock powder with suitable Fe/Ni ratio or by an alternative choice or modification of the substrate. The formation of a three-layer FGM from W–Cu mixtures was successfully demonstrated, while the maximum W content above 92 vol % was achieved.

While the properties of these claddings (namely the presence of intermetallic phases and the moderate thermal conductivity) do not qualify them for the application in PFCs of fusion reactors, the current experiments have demonstrated the feasibility of this technique for the formation of W-based composites with gradually varying composition and high W content. The abovementioned drawbacks can be overcome by a selection of suitable material combination in addition to W. Experiments in this direction are underway.

## Figures and Tables

**Table 1:** Characteristics of the used materials. For the steel substrate, the presented composition comes from X-ray fluorescence spectrometry (XRF) measurements and agrees well with the general specifications [25].

Material.	Size Range (μm)	d50 (μm)	Composition
W	65–110	80	W
Ni-340	90–180	146	Ni–4Cr–3Mo–1B–2.8Si–1.9P
Cu	130–210	174	Cu
AISI 303			71.4Fe–18.4 Cr–7.8 Ni–1.5Mn

**Table 2:** XRF results from the surface of W–Ni claddings on a steel substrate.

Sample	25% W	50% W	75% W
Element	(wt %)		
Cr	4.0	4.1	3.4
Fe	0.3	0.3	0.6
Ni	85	60.3	49.7
Mo	3	2.2	1.5
W	7.8	31.6	44.5

## Data Availability

Data is contained within the article.
